# Effect of Milling on Nutritional Components in Common and Zinc-Biofortified Wheat

**DOI:** 10.3390/nu15040833

**Published:** 2023-02-06

**Authors:** Zefang Jiang, Shiyue Zhou, Yu Peng, Xin Wen, Yuanying Ni, Mo Li

**Affiliations:** 1 College of Food Science and Nutritional Engineering, China Agricultural University, Beijing 100083, China; 2 National Engineering Research Center for Fruits and Vegetables Processing, Beijing 100083, China

**Keywords:** biofortified wheat, mill fractions, nutritional components, minerals, vitamin B, bioaccessibility

## Abstract

Biofortification is one of the most successful approaches to enhance the level of micronutrients in wheat. In the present study, wheats with zinc biofortification (foliar fertilization and breeding strategies) were milled into five components (whole flour, break flour, reduction flour, fine bran, and coarse bran) and their mineral content and nutritional components were evaluated. The results revealed that biofortification greatly increased the Zn concentration (by 30.58%–30.86%) and soluble Zn content (by 28.57%–42.86%) of whole flour after digestion. This improvement is mainly in break flour, reduction flour, and fine bran. Meanwhile, the contents of macronutrients including ash, lipids, and proteins and micronutrients containing iron, calcium, and vitamins (B_1_, B_6_, and B_9_) increased after biofortification. In addition, there was a decline in the concentrations of vitamins B_2_ and B_5_. Although dietary fibers and starch are the major carbohydrates, total dietary fiber exhibited a declining trend in coarse bran, and starch exhibited a rising trend in break and reduction flour. There was a decrease in the molar ratio of phytates: zinc did not promote a significant improvement in zinc bioaccessibility. These results can be useful for generating wheat varieties rich in micronutrients as well as having better nutritional traits.

## 1. Introduction

Zinc is a component of many proteins and enzymes, playing an important role in human immunity, catalysis, and biochemical [1]. Zinc deficiency in the human body can lead to impaired immune function as well as stunted growth in children. Zinc deficiency is widespread, with 82% of pregnant women worldwide intaking insufficient zinc, at least 25–40% of the population deficient in zinc, and ~50% at risk of zinc deficiency [2]. Zinc malnutrition can be addressed through strategies such as food fortification, zinc supplementation, and dietary diversification. However, these strategies are not always effective, immediate, feasible, and affordable. The intake of zinc through industrial products, including nutritional supplements, besides resulting in high costs, presents a higher risk of toxicity on account of excessive intake. Biofortification circumvents these problems by improving the zinc content of the crops themselves by its introduction in fertilization programs and by genetic improvement of cultivars, which is necessary to meet the human need, especially low-income populations. There are various methods of zinc application in agronomic biofortification, among which soil application and foliar application are the principal and conventional methods.

Wheat is the most widely planted crop in the world and is consumed by humans as a staple food. It is one of the most common carriers of biofortification and is considered a feasible solution to widespread human Zn deficiency in the developing world [3]. However, zinc tends to accumulate in the husks of grains such as bran and germ, but less in the inner endosperm. The majority of wheat products consumed in the diet are produced after grain milling, which removes most of the bran and germ, thereby reducing the amounts of minerals in wheat products. Even though biofortification might be achieved, the concentration of zinc in those products stemming from refined flour is significantly reduced [4]. Numerous studies have indicated that milling is a critical process affecting the amount of minerals in wheat products, and these amounts depend on grain shape and texture (which depend also on the cultivar), mineral distribution within the grain, and pearling or debranning degrees [5]. Therefore, it is important to consider the distribution of minerals within the grain after biofortification for evaluating the nutritional benefits of biofortified wheat products. 

Recently, more attention has been directed toward improving flour extraction or recycling the wheat bran produced by milling in terms of their nutritionally valuable minerals, hormonally active compounds, vitamins, and several antioxidant compounds (ferulic acid, coumaric acid, lutein, etc.). Improving the proportion of bran in wheat flour and its derivative products is an effective method for increasing the content of nutrients [6]. Meanwhile, whole-grain wheat flour, which is rich in minerals compared to refined flour, is also especially popular because of its ability to reduce the risks of coronary heart disease, regulate the level of blood glucose, and inhibit several forms of cancer [7]. However, whole-grain flour and bran contain significant amounts of mineral antinutrients (e.g., phytic acid, polyphenols, and dietary fibers), which affect Zn bioaccessibility and limit their use in food formulation. The mineral antinutrients can bind the minerals, reducing the solubility of Zn in food and potentially inhibiting Zn absorption by the human intestine [8]. For this reason, it is also useful to evaluate other nutritional components and Zn bioaccessibility in whole-wheat grain and wheat brans. Based on this collection of information, a suitable method can be applied to reduce the amount of mineral antinutrients, improve Zn bioaccessibility, and also retain the potential benefit to human health [4]. 

**Figure A1:**
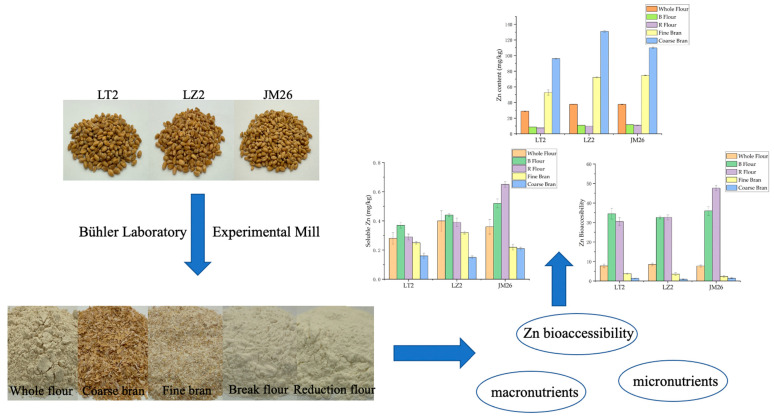
The conceptual figure of this study.

Against this background, in this study, zinc-fortified wheat with fertilization or breeding was selected to Bühler Laboratory Experimental Mill, and different milling fractions were obtained. The distribution of nutritional components in Zn-biofortified wheat and the effect of biofortification on Zn bioaccessibility were investigated. The resulting knowledge can be used to optimize Zn-biofortified wheat grain processing methodologies and provide more nutritional information to alleviate Zn deficiency by combining agricultural production with food processing (the conceptual figure was showed in Figure A1). 

## 2. Materials and Methods

### 2.1. Materials

The agronomic biofortified wheat variety (Longtang2-Zn, LZ2) was donated by the College of Resources and Environment Science, China Agricultural University, which was performed by foliar fertilization with a solution containing 0.4% ZnSO_4_·H_2_O (*w*/*v*). A field experiment was conducted in 2020–2021 at the Quzhou Experiment Station (36.9° N, 115.0° E) in China. Another wheat selected from breeding program was a high-zinc variety (Jimai26, JM26), which was donated by the College of Agronomy and Biotechnology, China Agricultural University. No agronomic biofortified wheat (Longtang2, LT2) was used as the control. All grains were stored at −20 °C before use. All chemicals, solvents, and reagents were of analytical grade and purchased from Sigma–Aldrich (Shanghai, China), unless otherwise stated.

### 2.2. Milling

The wheat samples were hand-cleaned and tempered to 14.0% moisture content. Then, the samples were milled using the Bühler Laboratory Experimental Mill (MLU-202) to obtain break streams flour, reduction streams flour, and two types of bran (coarse and fine) [9]. Whole flour contained all milling fractions (100% of the grain). The percentages of milling fractions are listed in Table 1. After milling, the five fractions (whole flour, coarse bran, fine bran, B flour, and R flour) were stored at room temperature (25 °C) and under low relative humidity (60%) in paper bags until analysis.

### 2.3. Analysis of Basic Nutrients 

Protein content was calculated using Kjeldahl’s method according to AACC 46-10 (Nx6.25). Lipids content was calculated using Soxhlet’s extraction according to AACC 30-10. Ash content was estimated according to AACC 08-01, in which a 0.5 g sample was kept in a Muffle furnace at 550 °C for 12–18 h after carbonization. Total dietary fiber (TDF), including soluble dietary fiber (SDF) and insoluble dietary fiber (IDF), was determined using the enzyme–gravimetric method according to AACC 32-07. Starch content was determined [10] and the total carbohydrates content was calculated using the following equation:

total carbohydrates = 100 − (% protein + % lipids + % ash + % moisture).(1)

The calorific value was calculated using the method described in AOAC (2015). The values obtained for proteins, lipids, and carbohydrates were used to calculate the calorific value of the sample as follows:

calorific value (kCal/(100 g)) = (proteins × 4.0) + (lipids × 9.0) + (carbohydrates × 3.75).(2)

### 2.4. Analysis of Mineral Contents 

Mineral element contents were determined using an inductively coupled plasma–optical emission spectrophotometer (ICP-OES, Jobin Yvon Horiba ULTIMA 2C). The samples were pretreated with wet digestion before instrument measurement [11]. Briefly, 1 g grain sample or 5 mL liquid samples of in vitro digestion were removed from the digestion vessel, and a 10 mL mixture of nitric acid and perchloric acid (10:1) was added, until white smoke appeared. The digestive solution was transparent or slightly yellow. It was cooled and diluted to 25 mL with deionized water. 

### 2.5. Analysis of Phenolic Content

First, phenol from grain was extracted, and then the content of each sample was measured using a Folin–Ciocalteu reagent [12]. Acidic methanol (20 mL of 50% (*v*/*v*)) was added to the sample, and the sample was shaken for 60 min and centrifuged at 2500× *g* to extract the polyphenols. The residue was extracted using 20 mL of 70% (*v*/*v*) acetone one more time, and the supernatants were combined. Folin–Ciocalteu reagent (5 mL) and 8 mL of 15% sodium carbonate were added to the mixed solution, respectively. After incubation for 120 min, the absorbance was measured at 765 nm. The total phenolic content in cereals was calculated according to the corresponding concentration of the standard curve.

### 2.6. Analysis of Phytic Acid Content

Phytic acid content was measured by using the colorimetric procedure [13]. In total, 0.1 g of sample was taken and 10 mL of 0.2 mol/L HCl was added for extraction for 2 h. After centrifugation at 5000× *g*, 0.5 mL of supernatant was removed, to which 1 mL 0.02% FeNH_4_(SO_4_)_2_ was added, and the solution was kept in a boiling water bath for 30 min. Then, 2 mL of 1% C_10_H_8_N_2_–C_2_H_4_O_2_S was added. After mixing, the absorbance was measured at 519 nm. The molar ratios of Phy:Fe and Phy:Zn were determined by dividing the moles of phytic acid by the moles of minerals in a 100 g sample.

### 2.7. Analysis of Vitamin B Contents 

Vitamins B_1_ (thiamine), B_2_ (riboflavin), B_5_ (pantothenic acid), B_6_ (pyridoxine), and B_9_ (folic acid) were quantified. Briefly, a 5 g sample was weighed and 60 mL of 0.1 mol/L HCl was added. This solution was then incubated at 121 °C for 30 min and cooled to below 40 °C. The pH was adjusted to ~4.0 with 2.0 mol/L CH_3_COONa, and 2.0 mL of enzyme solution was mixed in and the resulting solution was placed in an incubator at 37 °C overnight (for ~16 h). The solution was diluted in distilled water to 100 mL, and the supernatant was taken after centrifugation. The derivatized solution was filtered through a 0.45 μm Millipore filter. A reversed-phase high-performance liquid chromatography system with a C18 column (measuring 250 mm × 4.6 mm, with a pore size of 5 μm) was used. The mobile phase of CH_3_COONa–CH_3_OH (65 + 35) had a flow rate of 0.8 mL/min. The injection volume was 20 μL and the excitation and emission wavelengths were 375 and 435 nm, respectively [14].

### 2.8. Estimation of Zn Bioaccessibility by In Vitro Gastrointestinal Digestion 

Static in vitro digestion was performed according to INFOGEST 2.0 [15]. The activities of various enzymes in the model were measured. For the oral phase, a 5 g sample was prepared into a paste, and 3 mL of simulated salivary fluid and 1 mL of α-amylase solution (75 U/mL) were added to the sample. The mixture was shaken (at 37 °C and 95 rpm for 2 min). For the gastric phase, 6 mL of simulated gastric fluid was to the digestive solution and the pH was adjusted to 4. Then, 2 mL of pepsin solution (2000 U/mL) and gastric lipase (60 U/mL) were added, and the pH was adjusted to 3. The mixture was overlain with nitrogen and shaken (at 37 °C and 95 rpm for 2 h). For the intestinal phase, the pH of digestive solution was adjusted to 6, then 5 mL of bile (10 mmol/L) and 10 mL of pancreatin (with a trypsin activity of 100 U/mL) were added, after which the pH was adjusted to 7. The mixture was overlain with nitrogen and shaken (at 37 °C and 95 rpm for 2 h). After centrifugation and filtration, the concentration of soluble elements (in mg/kg) in in vitro gastrointestinal digestion was analyzed using ICP-OES. Control experiments were performed, and all assays were performed in triplicate (*n* = 3). Bioaccessibility (BAC) was calculated according to the following formula:

BAC (%) = BE/TE,(3)

where BE = fraction of bioaccessible element (in mg) and TE = total element content (in mg) in samples. 

### 2.9. Statistical Analysis 

SPSS 22.0 was used for the statistical analyses. Tukey’s test was used to determine significant differences. All data were analysis of variance data determined by the least significant differences test at *p = 0.05*. All experiments were performed in three independent trials, and the results were expressed as the mean ± standard deviation.

## 3. Results and Discussion

### 3.1. Basic Nutritional Components of Different Wheat Samples

The basic nutritional components of milling fractions of LT2, LZ2, and JM26 are presented in Table 2. The ash content of milling fractions of the three wheat samples ranged from 0.34 to 5.36 g/100 g, among which coarse bran had the highest content (4.96–5.36 g/100 g). Biofortified wheat exhibited a different distribution of ash content in milling fractions from that of common wheat (*p* < 0.05). The whole flour ash content of LZ2 (1.52 g/100 g) was higher than that of LT2 (1.30 g/100 g), but had no difference from that of JM26 (1.53 g/100 g). Ash corresponds to mineral content, and this indicates that the mineral content of wheat was improved after application of zinc fertilizer. This is in line with the conclusion that fertilization increased ash content compared with the control [16]. Compared with LT2, LZ2 exhibited no significant difference in the ash content of B flour and R flour, but decreased content of ash in fine bran. The increase in ash content in LZ2 (5.36 g/100 g) was mainly concentrated in coarse bran, being higher than that in LT2 (5.03 g/100 g). Among the milling fractions of JM26, B flour, R flour, and fine bran had higher ash content than that of LT2 and LZ2, being 0.55, 0.53, and 2.58 g/100 g, respectively, but coarse bran had lower ash content. The lipids distributions in different milling fractions in the three wheat varieties were similar. The lipids content of fine bran was the highest, being 2.48 (LT2), 2.90 (LZ2), and 3.09 g/100 g (JM26), respectively, followed by that of coarse bran and whole flour, with the least amount in B flour and R flour. During the grinding process, the lipids-rich germ is distributed into the fine bran [17]. The lipids content of each milling fraction of JM26 was the highest among the three wheat varieties. After zinc foliar fertilization, the lipids content of LZ2 whole flour (1.54 g/100 g) was significantly increased compared with that of LT2 (1.28 g/100 g) (*p* < 0.05). Previous studies reported a 25.8% increase in grain lipids content after foliar fertilization [18]. The increased content of lipids in flour creates a smoother surface of starch granules and can improve dough rheological properties and wheat flour quality.

High protein has a significant effect on improving the quality of wheat products, promoting excellent viscoelasticity, extensibility, and water absorption. After zinc fertilization, the protein content of LZ2 (12.75 g/100 g) was higher than that of LT2 (10.00 g/100 g) and similar to that of JM26 (12.02 g/100 g). This is consisted with the previous conclusion that suggested a significant positive correlation between zinc and protein content in wheat grains [19]. The increase of protein may improve wheat’s ability to accumulate zinc, because protein provides an important reservoir for zinc accumulation in grains. In the milling fractions of all three wheat samples, protein content was the highest in the coarse bran (14.42–16.00 g/100 g), followed by B flour (12.19–13.94 g/100 g), and fine bran (11.74–13.02 g/100 g), with the lowest content in R flour (9.55–11.03 g/100 g). As was previously described, the protein content was the highest in the aleurone layer and the lowest in the endosperm layer [20].

As presented in Table 2, the three species of wheat contained comparable amounts of total carbohydrates, ranging between 70.84 and 73.42 g/100 g. Similar results in wheat flour were reported in whole-grain wheat (70.90 g/100 g) from Poland [21]. The carbohydrate contents of B flour (70.93–72.68 g/100 g) and R flour (73.53–75.27 g/100 g) differed little from that of the whole flour (*p* > 0.05), and the carbohydrate content of coarse bran (61.88–64.03 g/100 g) was the lowest. The variation in carbohydrates was interesting; namely, there were similar amounts of carbohydrates in different wheat types of the same milling fractions, whereas dietary fibers and starch, which are the major carbohydrates, exhibited differences in wheat grains. 

TDF (including SDF and IDF), which binds with minerals to form complexes by electrostatic force or chelation, can be considered as a mineral antinutrient [22]. The TDF, IDF, and SDF contents in the three wheat varieties are given in Table 2. The TDF content in B flour (1.26–1.31, g/100 g) and R flour (1.28–1.31, g/100 g) were the lowest, the content in fine bran (15.95–17.73 g/100 g) was higher than that in whole flour (10.95–11.81 g/100 g), and the content in coarse bran (39.36–44.47 g/100 g) was the highest. SDF and IDF also exhibited the same trend. Research results are in agreement with earlier findings that the TDF, IDF, and SDF contents of wheat bran were in the ranges of 36.5–52.4, 35.0–48.4, and 1.5–4.0 g/100 g, respectively [23]. Comparison with LT2 showed that there was no significant difference in TDF contents among whole flour, B flour, and R flour of LZ2 and JM26 (*p* > 0.05), while JM26 had a higher TDF content in fine bran (17.73 g/100 g) than that in LT2 fine bran (16.62 g/100 g) and LZ2 fine bran (15.95 g/100 g), while that of coarse bran was lower. A significant difference in SDF of whole flour was observed (*p* < 0.05): SDF content of JM26 (1.69 g/100 g) was the highest, followed by LZ2 (1.20 g/100 g) and LT2 (0.94 g/100 g). The fermentation of SDF in the colon contributes to colonic microbiota producing short-chain fatty acids and an acidic environment, which promotes epithelial cell proliferation, thereby increasing the absorptive surface area for utilization of minerals [8]. The contents of IDF in B flour and R flour in the three wheat samples were between 0.81 and 0.82 g/100 g. The coarse bran content of LT2 (40.82 g/100 g) was close to that of LZ2 (41.55 g/100 g), both being higher than that of JM26 coarse bran (36.75 g/100 g).

As shown in Table 2, in all wheat varieties, the starch contents of B flour (72.60–75.53 g/100 g) and R flour (74.10–76.14 g/100 g) were significantly higher than those of fine bran (52.79–58.65 g/100 g) and coarse bran (16.78–23.14 g/100 g) (*p* < 0.05). After application of zinc fertilizer, the whole flour starch content of LZ2 (66.20 g/100 g) was higher than that of LT2 (63.40 g/100 g). Studies have reported that application of Zn fertilizer markedly enhanced the amount of starch in the wheat [24]. This may be explained by the fact that Zn contributes to photosynthesis, chlorophyll formation, and the metabolism of starch formation. The increase in starch content was mainly reflected in B flour and R flour, with values of 75.53 and 76.10 g/100 g, respectively, for LZ2 and 72.60 and 74.20 g/100 g, respectively, for LT2. The coarse bran of JM26 had a higher starch content than that of LT2 and LZ2, which was related to wheat breeding and planting conditions. According to the above data, coarse bran had the lowest amount of starch and the highest amount of TDF, which limited the processability of whole-grain foods. In terms of energy, in all wheat varieties, the calorific values of all milling fractions were in the range of 302.47–332.64 kcal/100 g. Coarse bran exhibited the lowest calorific value in all milling fractions, because coarse bran is lower in carbohydrates and lipids, while there was no statistical difference among other milling fractions (*p* > 0.05). 

### 3.2. Vitamin B Contents of Different Wheat Samples

Different wheat samples contain several B vitamins, such as VB_1_, VB_2_, VB_5_, VB_6_, and VB_9_. These have many positive effects on human metabolism, including fat, protein, and carbohydrate metabolism, thus benefiting human health. As shown in Table 3, the VB_1_ content of whole flour ranged from 1.39 to 1.93 μg/g in the three wheat varieties. The highest amount of VB_1_ was found in fine bran (2.65–3.89 μg/g). Among the three wheat samples, JM26 had the highest VB_1_ content in whole flour and each milling fraction. Compared with LT2, LZ2 had a higher VB_1_ content in whole flour (1.56 μg/g) and fine bran (3.89 μg/g), representing increases of 12.2% and 6.3%, respectively. Except for VB_1_, the highest contents of VB_2_, VB_5_, VB_6_, and VB_9_ were detected in coarse bran, followed by fine bran and whole flour. VB_5_ was found to be the most abundant B vitamin in different milling fractions of selected wheat samples, with values varying from 18.17 to 38.21 μg/g for coarse bran, from 2.62 to 4.96 μg/g for whole flour, and from 0.98 to 3.29 μg/g for B flour and R flour. It was reported that the VB_5_ content was significantly higher in coarse bran (32.28 μg/g) than in the other fractions (3.61–20.08 μg/g) (*p* < 0.05) [25]. Compared with LT2 whole flour (3.14 μg/g), there was a decline of VB_5_ in LZ2 (2.62 μg/g), mainly in R flour (0.98 μg/g) and coarse bran (18.17 μg/g), with decreases of 54.0% and 22.9%, respectively. Regarding the VB_2_ and VB_6_ contents, coarse bran had the highest amount (7.09–7.60 and 3.56–5.21 μg/g, respectively), followed by whole flour (0.44–0.61 and 1.21–1.64 μg/g, respectively), while R flour had the lowest amount (0.15–0.19 and 0.42–0.77 μg/g, respectively) of the three wheat varieties. The application of zinc fertilizer reduced the content of VB_2_ in whole flour by 27.9%. Specifically, the contents in fine bran and coarse bran decreased by 38.3% and 7.1%, respectively. However, zinc fertilizer increased the content of VB_6_ in B flour and R flour, and the growth was found to be 159.5% and 83.3%, respectively. The high-zinc wheat variety (JM26) had lower VB_2_ and higher VB_6_ contents, when compared with those of LT2. The VB_9_ content accounted for the lowest proportion among these several B vitamins. Previous studies have also reported lower VB_9_ contents of wheat flour and grains (10.14–11.92 μg/100 g and 32.35–42.67 μg/100 g, respectively) [26]. In terms of the milling fractions, the contents of VB_9_ were also the highest in coarse bran, with values of 112.20–225.80 μg/100 g. After biofortification, there were significant differences among LT2 (68.44 μg/100 g), LZ2 (89.75 μg/100 g), and JM26 (115.10 μg/100 g) (*p* < 0.05). The contents of VB_9_ in LZ2 and JM26 increased by 31.1% and 68.2% compared with those in LT2. The contents of VB_9_ in B flour and R flour increased from 20.75 and 24.11 μg/100 g to 48.57 and 42.35 μg/100 g, respectively, after zinc fertilization.

### 3.3. Mineral Composition of Different Wheat Samples

In terms of the distribution of zinc, the highest amount was registered for coarse bran (96.07–130.95 mg/kg), followed by fine bran (52.72–74.64 mg/kg) and whole flour (28.81–37.70 mg/kg). The other two milling fractions exhibited a very similar quantity of Zn, varying from 8.58 to 11.57 mg/kg for B flour and from 7.60 to 10.92 mg/kg for R flour. Similar observations were also reported [9]. As shown in Table 4, the aim of biofortification was realized; that is, zinc concentration was significantly increased (*p* < 0.05). When Zn fertilizer was applied, the Zn concentration of LZ2 (37.70 mg/kg) was increased by 30.86%. Similarly, the zinc concentration of JM26 also reached 37.62 mg/kg. A positive response to biofortification has also been already reported for many other wheat varieties [27]. Foliar application of 0.67% ZnSO_4_·H_2_O (*w*/*v*) increased grain Zn concentration by 28%−68% depending on the variety [28]. Importantly, the increase after biofortification is reflected in each milling fraction. First, the growth in the concentrations in B flour and R flour were 25.9%–34.8% and 27.0%–43.7%, respectively, which is quite valuable, because the human body mainly absorbs this part of Zn. In addition, the content of Zn had the highest increase in fine bran, with growth proportions of 37.0% and 41.6%, respectively. The Zn content in coarse bran was still higher than that in the rest of the milling fractions. Therefore, bran exhibited the highest potential for improving zinc content in cereal products. Generally, zinc located in epidermal cells, mesophyll cells, and vascular parenchyma cells is pumped into the apoplast space via heavy metal-associated proteins, then loaded into the phloem via ZRT-/IRT-like proteins (ZIPs) and yellow-stripe-like-family (YSL) proteins, and finally transported to the grains through the phloem [29]. The transport of zinc from the seed coat to the endosperm is hampered by a number of factors, which result in low levels of zinc in the endosperm. These include insoluble ligands with phytic acid in the aleurone layer, multiple transporters and chelating molecules localized in the outer tissue region, and the amount and activity of transporters [30]. 

The major quantity of Ca was found in coarse bran (982.17–1085.24 mg/kg). B flour exhibited higher Ca contents (240.83–250.87 mg/kg) compared to that in R flour, which was the milling fraction with the least amount of Ca (208.33–234.90 mg/kg). Of the three wheat samples, the higher amount was found for JM26 and LZ2 of whole flour (365.60 and 364.64 mg/kg, respectively). Compared with LT2 (343.52 mg/kg), the Ca content increased by ~6%. With zinc fertilization, LZ2 had Ca contents of 234.90 and 1085.24 mg/kg in R flour and coarse bran, respectively, which are significantly higher than those of LT2 (222.39 and 982.17 mg/kg, respectively) (*p* < 0.05). As was previously reported, the application of zinc fertilizer increased the calcium content of wheat grain [31]. For the Fe content of different milling fractions, the results were 8.1–11.35 and 111.72–138.58 mg/kg in R flour and coarse flour, respectively. The Fe content in whole flour was in the range of 36.10–39.30 mg/kg. Guttieri et al. reported that Fe accumulated primarily in the outermost layer of the bran and that the Fe content in straight grade flour was only 8% of the content in bran [32]. After zinc biofortification, significant differences in Fe contents were detected between LZ2 (JM26) and LT2, which were mainly reflected in coarse bran and B flour (*p* < 0.05). Fe contents in LZ2 and JM26 increased by 35.8% and 11.8%, respectively, in B flour and by 24.2% and 18.4%, respectively, in coarse bran. This is consistent with the results of previous studies that demonstrated that Fe and Zn contents were highly positively correlated [33]. This phenomenon suggests that iron and zinc have something in common as transporters or absorption pathways, also suggesting that high Zn content can be accompanied by high contents of some other minerals [34]. 

### 3.4. Polyphenol and Phytate Contents of Different Wheat Samples

As presented in Table 5, biofortification with Zn was not shown to increase the accumulation of total polyphenols in whole flour. Phytates content in LZ2 (15.99 mg/g) was not significantly different from that in LT2 (15.22 mg/g) (*p* > 0.05), and that in JM26 was found to be lower (12.47 mg/g) than that in LT2 and LZ2, which directly results from the genotype and plant growth conditions. Moreover, no significant difference was found in the same milling fractions among the three wheat samples (*p* > 0.05). It was also reported that the total amount of polyphenols in whole flour did not change significantly, nor did the antioxidant activity, after foliar Fe and Zn biofortification [35]. In different milling fractions, coarse bran had the highest concentration of phytates. LZ2 had the highest content of phytates in coarse bran (54.47 mg/g), with LT2 and JM26 having lower contents (47.88 and 39.33 mg/g, respectively). Therefore, no uniform conclusion can be reached about the effect of zinc biofortification on phytates content. Some authors suggest that the phytates concentration in the flours and bran was generally not affected by Zn biofortification [9]. Other researchers have reported that agronomic zinc biofortification resulted in a reduction of phytates in wheat grains [36]. 

The molar ratios of phytates and mineral are a common indicator for assessing the potential bioavailability of mineral elements. The higher the molar ratio is, the lower is bioavailability of the mineral. Molar ratios of Phy:Zn < 15 and Phy:Fe < 1 significantly improve Zn and Fe absorption, whereas molar ratios of Phy:Ca < 0.17 have been associated with Ca bioavailability. The obtained results indicated that coarse bran had higher values of Phy:Zn (35.26–49.08) and Phy:Fe (25.25–36.36) molar ratios, as well as Phy:Ca (2.39–3.04). Although coarse bran was found to be a good source of some minerals, such as Zn, Fe, and Ca, the significant content of phytates limits their bioavailability. B flour and R flour had the lowest values of Zn, Fe, and Ca (with ratios of 11.72–16.67, 5.02–12.86, and 0.30–0.43, respectively). Zhang et al. found that the value of Phy:Zn in bran was higher than that in other fractions [37]. It is noteworthy that whole flour also had relatively unfavorable molar ratio values, with Phy:Zn of 32.64–52.50 and Phy:Fe of 26.99–36.09, but had no significant difference from that of coarse bran (*p* > 0.05). This needs to be considered in promoting whole-grain foods, which are rich in nutrients as well as antinutritional factors. As expected, the values of Phy:Zn were obviously influenced by Zn biofortification. We observed that LZ2 and JM26 had lower molar ratios in comparison to LT2 in both whole flour and each milling fraction. The molar ratio in B flour and R flour was <15, while for the control sample, it was >15, and the decline in other milling fractions reached 16.5%–37.8%. Zhao et al. also reported that foliar Zn application can decrease the value of Phy:Zn by 36% and improve the bioavailability of Zn in wheat flour and whole grain [38]. It may be assumed, given the content of investigated antinutrients, that wheat samples with Zn biofortification are a valuable source of Zn with relatively high concentration and good bioavailability of this element. In addition to Phy:Zn, biofortification with Zn slightly reduced the values of Phy:Ca and Phy:Fe only in the B flour, R flour, and coarse bran.

During food processing, phytates content can be degraded when phytases are activated at optimal technological conditions. Therefore, it is necessary to understand the phytase activity in wheat flour. In our study, no difference was observed among the three wheat samples (*p* > 0.05). The phytase activity in B flour (0.27–0.29 U/g) and R flour (0.19–0.25 U/g) was very similar, both being significantly lower than that in whole flour (0.41–0.48 U/g), coarse bran (0.80–0.87 U/g), and fine bran (0.54–0.59 U/g) (*p* < 0.05). Our results are consistent with previous studies that indicated that wheat grain phytase activity decreased along with pearling extension from the outer to inner layer [39], because coarse bran had the maximum phytase activity. Therefore, it is anticipated that the endogenous phytase activity of coarse bran can be activated to reduce phytate content using appropriate processing technology, thereby increasing the bioavailability of micronutrients. This could improve the application potential of coarse bran.

### 3.5. Bioaccessibility of Zinc in Different Wheat Samples

**Figure 1:**
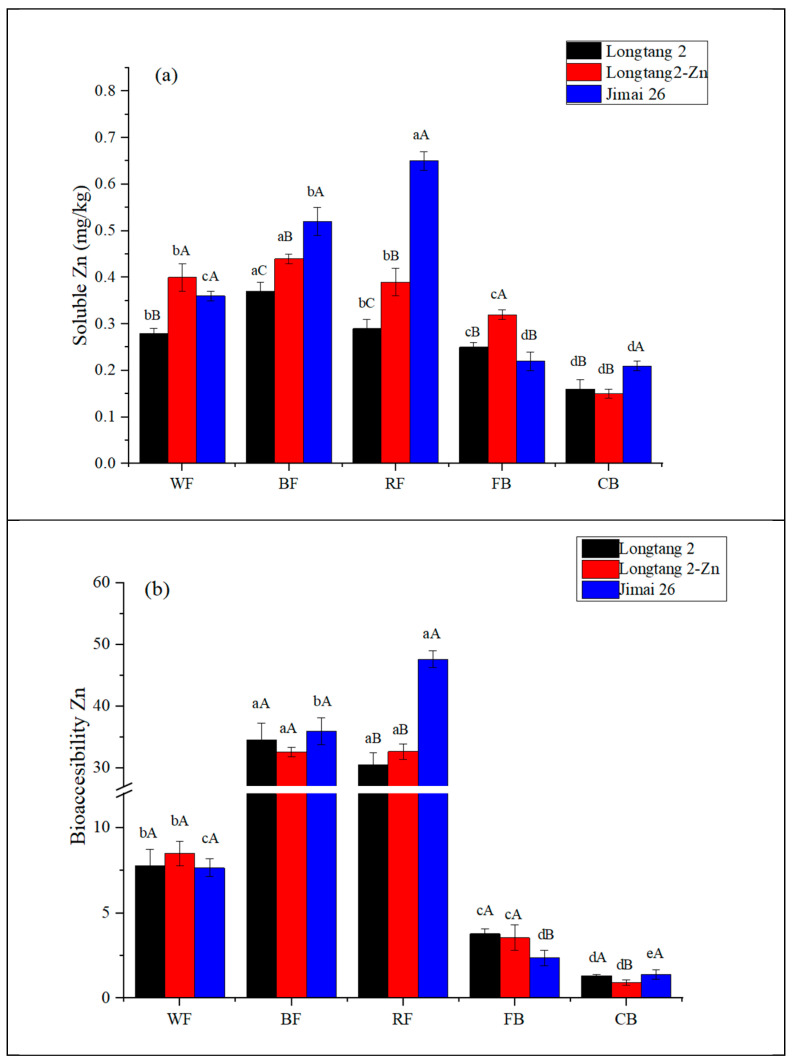
(**a**) Soluble Zn and (**b**) bioaccessibility of Zn in milling fractions of LT2, LZ2, and JM26. Longtang 2 (LT2) was used as the control sample; Longtang 2 with foliar fertilization will be referred to as Longtang 2-Zn (LZ2); the other selected wheat Jimai26 (JM26) was a high-zinc variety. WF: whole flour; BF: B flour; RF: R flour; FB: fine bran; CB: coarse bran. Data are expressed as mean ± standard deviation (*n* = 3). For the same parameter, means with different superscript lowercase letters within different milling fractions of the same wheat species are significantly different (*p* < 0.05), and means with different superscript uppercase letters between within different wheat samples of the same milling fractions are significantly different (*p* < 0.05).

According to the above analysis, Zn content in both whole flour and various milling fractions was increased by biofortification, but whether the increased Zn content can be absorbed and utilized by the human body remains an important problem. A widely used in vitro digestion model was employed to assess mineral bioaccessibility. The results related to the contents of soluble fractions and the bioaccessibility for Zn are shown in Figure 1. B flour and R flour were significantly higher in their soluble Zn values than the other milling fractions, ranging from 0.29 to 0.65 mg/kg (*p* < 0.05). The soluble Zn in whole flour (0.28–0.40 mg/kg) exhibited lower values and the coarse bran had the lowest values (0.15–0.21 mg/kg,). Bioaccessibility also exhibited the same pattern as the content of soluble zinc. B flour and R flour had the highest bioaccessibility (30.52%–47.62%), followed by whole flour (7.65%–8.49%), with the lowest being coarse bran (0.92%–1.40%). This is in line with previous results for whole flour ranging from 2.5% to 8.9% [40,41]. It was reported that the bioaccessibility of Zn was higher for white flour pasta than for whole-wheat pasta [42]. These results suggest that, although coarse bran and whole flour had higher Zn concentrations, after in vitro digestion, the bioaccessibility of Zn, on average, was higher for white flour.

After zinc biofortification, regardless of fertilization or breeding methods, soluble Zn content was increased by 18.92%–21.43% in B flour, 34.48%–124.14% in R flour, and 28.57%–42.86% in whole flour, respectively. This is consistent with our previous results for the molar ratio of Phy:Zn, which exhibited lower values with treatment of biofortification in comparison to those of LT2, being conducive to zinc absorption. This demonstrates that such measures of biofortification make sense, as obtained zinc can be transported into the human gut and be ready to be absorbed after digestion. No statistical difference was observed in the Zn bioaccessibility of whole flour and B flour in the three wheat samples (*p* > 0.05), being 7.78% and 34.50%, 8.49% and 32.59%, and 7.65% and 35.96% for LT2, LZ2, and JM26, respectively. Only in JM26 was the bioaccessibility of R flour higher, reaching 47.62%, and that of whole flour of LZ2 had a slightly higher value (8.49%). The zinc content was increased by biofortification, reducing the molar ratio of Phy:Zn and achieving higher amounts of soluble zinc after digestion. However, during the digestion process, the transport of Zn from ingestion to intestine reduces Zn content and changes its chemical form, which may not be conducive to zinc absorption. In addition, wheat flour still contains high contents of other antinutritional factors, such as fibers and phytates, and these can inhibit the release of minerals from wheat milling fractions [43]. Improving bioaccessibility requires a combination of biofortification and food processing, such as fermentation, germination, or other emerging techniques.

## 4. Conclusions

The development of wheat species with improved levels of micronutrients is one of main targets in biofortified cereals programs. The presentation of nutritional characteristics of biofortified wheat varieties not only provides an important reference for the development of Zn-rich biofortified wheat with high nutritional value, but also optimizes the food supply scheme to overcome micronutrient deficiencies. The present study reveals that biofortified wheat not only had higher concentrations of zinc (increase by 30%), but also increased contents of soluble Zn (increase by 28.57%–42.86%) after digestion, while there was no significant effect on the amount of bioaccessible zinc. Differences in macronutrients (ash, lipids, proteins, and starches) and micronutrients (Fe, Ca, and B vitamins) were observed after biofortification. Meanwhile, the variable responses of nutritional components in milling fractions should be taken into consideration for introducing biofortification strategies. Biofortification has led to a substantial increase in the zinc content of B flour (25.9%–34.8%), R flour (27.0%–43.7%), and fine bran (37.0%–41.6%), and the difference in dietary fiber was reflected in coarse bran and fine bran. B vitamins exhibited various changes in each milling fraction, whereas biofortification had little effect on the amounts of polyphenols, phytic acid, and carbohydrates. Combining biofortification and food processing technology is a crucial step to improving human micronutrient absorption from the perspective of the food supply chain.

## Figures and Tables

**Table 1:** Percentages of milling fractions (B flour, R flour, coarse bran, and fine bran) and whole flour of LT2, LZ2, and JM26.

Wheat Variety	B Flour	R Flour	Fine Bran	Coarse Bran	Whole Flour
LT2	18.7	58.3	10.6	12.4	100
LZ2	20.2	56.5	11.6	11.7	100
JM26	15.6	56.2	13.2	15.0	100

Longtang 2 (LT2) was used as the control sample; Longtang 2 with foliar fertilization will be referred to as Longtang 2-Zn (LZ2); the other selected wheat Jimai26 (JM26) was a high-zinc variety.

**Table 2:** Basic chemical composition (g/100 g, with calorific value represented as kcal/100 g) of milling fractions of LT2, LZ2, and JM26.

Wheat Sample	Fraction	Ash	Lipids	Proteins	Starch	TDF	SDF	IDF	Carbohydrates	Calorific Value
LT2	Whole flour	1.30 ± 0.03 ^cB^	1.28 ± 0.09 ^cB^	10.00 ± 0.21 ^cB^	63.40 ± 0.63 ^cB^	10.95 ± 0.20 ^cA^	0.94 ± 0.03 ^cC^	10.01 ± 0.17 ^cB^	73.42 ± 0.13 ^aA^	326.85 ± 4.66 ^abA^
	B flour	0.40 ± 0.03 ^dB^	0.64 ± 0.04 ^eC^	12.48 ± 0.64 ^bAB^	72.60 ± 0.57 ^dB^	1.31 ± 0.01 ^dA^	0.49 ± 0.01 ^dA^	0.82 ± 0.01 ^dA^	72.68 ± 0.28 ^aA^	326.43 ± 1.83 ^bA^
	R flour	0.48 ± 0.05 ^dAB^	0.70 ± 0.02 ^dB^	9.55 ± 0.50 ^cB^	74.20 ± 0.65 ^dB^	1.28 ± 0.00 ^eA^	0.47 ± 0.02 ^dA^	0.81 ± 0.02 ^dA^	75.27 ± 2.53 ^aA^	326.76 ± 2.07 ^bA^
	Fine bran	2.31 ± 0.09 ^bB^	2.48 ± 0.11 ^aB^	12.85 ± 1.25 ^bA^	58.65 ± 0.56 ^bA^	16.62 ± 0.18 ^bB^	1.56 ± 0.04 ^bB^	15.06 ± 0.14 ^bA^	68.36 ± 0.77 ^bA^	330.07 ± 0.58 ^aA^
	Coarse bran	5.03 ± 0.03 ^aB^	1.82 ± 0.19 ^bB^	14.42 ± 0.36 ^aA^	19.78 ± 0.35 ^aB^	43.49 ± 0.66 ^aA^	2.68 ± 0.11 ^aB^	40.82 ± 0.77 ^aA^	64.03 ± 1.05 ^cA^	302.47 ± 2.41 ^cB^
LZ2	Whole flour	1.52 ± 0.04 ^cA^	1.54 ± 0.02 ^cA^	12.75 ± 0.47 ^bcA^	66.20 ± 0.52 ^cA^	11.81 ± 0.33 ^cA^	1.20 ± 0.01 ^cB^	10.61 ± 0.32 ^cA^	71.94 ± 2.33 ^bAB^	327.64 ± 2.70 ^aA^
	B flour	0.34 ± 0.01 ^dB^	0.79 ± 0.03 ^cdB^	13.94 ± 0.71 ^bA^	75.53 ± 0.88 ^dA^	1.26 ± 0.02 ^dA^	0.44 ± 0.01 ^dA^	0.82 ± 0.01 ^dA^	70.93 ± 0.57 ^bA^	328.86 ± 3.29 ^aA^
	R flour	0.34 ± 0.04 ^dB^	0.77 ± 0.04 ^cdB^	10.55 ± 1.06 ^dAB^	76.10 ± 0.59 ^dA^	1.28 ± 0.04 ^dA^	0.46 ± 0.02 ^dA^	0.82 ± 0.01 ^dA^	74.34 ± 0.36 ^aA^	327.91 ± 1.64 ^aA^
	Fine bran	2.14 ± 0.05 ^bC^	2.90 ± 0.18 ^aA^	11.74 ± 0.27 ^cdA^	56.81 ± 1.07 ^bB^	15.95 ± 0.45 ^bB^	1.50 ± 0.07 ^bB^	14.45 ± 0.38 ^bB^	69.22 ± 1.49 ^bA^	332.64 ± 5.51 ^aA^
	Coarse bran	5.36 ± 0.03 ^aA^	1.85 ± 0.12 ^bB^	16.00 ± 2.04 ^aA^	16.78 ± 0.33 ^aC^	44.47 ± 0.50 ^aA^	2.93 ± 0.12 ^aA^	41.55 ± 0.37 ^aA^	62.89 ± 0.50 ^cB^	306.59 ± 3.21 ^bB^
JM26	Whole flour	1.53 ± 0.01 ^cA^	1.61 ± 0.01 ^cA^	12.02 ± 0.58 ^bcA^	64.60 ± 0.79 ^cB^	11.33 ± 0.22 ^cA^	1.59 ± 0.04 ^cA^	9.74 ± 0.26 ^cB^	70.84 ± 1.31 ^aB^	328.22 ± 1.81 ^aA^
	B flour	0.55 ± 0.07 ^dA^	0.90 ± 0.05 ^dA^	12.19 ± 0.77 ^bcB^	73.96 ± 0.62 ^dB^	1.31 ± 0.03 ^dA^	0.49 ± 0.01 ^dA^	0.82 ± 0.02 ^dA^	72.36 ± 3.96 ^aA^	328.21 ± 1.27 ^aA^
	R flour	0.53 ± 0.11 ^dA^	0.91 ± 0.08 ^dA^	11.03 ± 0.57 ^cA^	76.14 ± 0.49 ^dA^	1.31 ± 0.02 ^dA^	0.49 ± 0.01 ^dA^	0.82 ± 0.01 ^dA^	73.53 ± 0.72 ^aA^	328.05 ± 1.53 ^aA^
	Fine bran	2.68 ± 0.03 ^bA^	3.09 ± 0.08 ^aA^	13.02 ± 0.52 ^bA^	52.79 ± 0.55 ^bC^	17.73 ± 0.23 ^bA^	2.00 ± 0.12 ^bA^	15.23 ± 0.11 ^bA^	67.21 ± 0.38 ^bA^	331.93 ± 5.28 ^aA^
	Coarse bran	4.96 ± 0.02 ^aC^	2.32 ± 0.06 ^bA^	14.84 ± 1.20 ^aA^	23.74 ± 0.50 ^aA^	39.36 ± 0.25 ^aB^	2.61 ± 0.16 ^aB^	36.75 ± 0.40 ^aB^	61.88 ± 0.55 ^cB^	312.29 ± 3.60 ^bA^

Data are expressed as mean ± standard deviation (*n* = 3). Longtang 2 (LT2) was used as the control sample; Longtang 2 with foliar fertilization will be referred to as Longtang 2-Zn (LZ2); the other selected wheat Jimai26 (JM26) was a high-zinc variety. TDF: total dietary fiber; IDF: insoluble dietary fiber; SDF: soluble dietary fiber. For the same parameter, means with different superscript lowercase letters within different milling fractions of the same wheat species are significantly different (*p* < 0.05), and means with different superscript uppercase letters within different wheat samples of the same milling fractions are significantly different (*p* < 0.05).

**Table 3:** Vitamin B contents (μg/g) of the milling fractions of LT2, LZ2, and JM26.

Wheat Sample	Fraction	VB_1_	VB_2_	VB_5_	VB_6_	VB_9_ (µg/100 g)
LT2	Whole flour	1.39 ± 0.04 ^bC^	0.61 ± 0.02 ^cA^	3.14 ± 0.14 ^cB^	1.21 ± 0.06 ^cB^	68.44 ± 1.36 ^cC^
	B flour	0.38 ± 0.02 ^eB^	0.18 ± 0.00 ^dA^	1.33 ± 0.07 ^eB^	0.42 ± 0.03 ^dC^	20.75 ± 0.94 ^eC^
	R flour	0.73 ± 0.04 ^dB^	0.15 ± 0.01 ^eA^	2.13 ± 0.11 ^dB^	0.42 ± 0.02 ^dB^	24.11 ± 1.11 ^dC^
	Fine bran	3.66 ± 0.01 ^aB^	1.07 ± 0.02 ^bA^	10.00 ± 0.52 ^bB^	2.76 ± 0.06 ^bA^	120.85 ± 2.33 ^bA^
	Coarse bran	1.01 ± 0.07 ^cB^	5.21 ± 0.34 ^aA^	23.57 ± 0.10 ^aB^	7.09 ± 0.45 ^aA^	150.45 ± 0.49 ^aB^
LZ2	Whole flour	1.56 ± 0.05 ^bB^	0.44 ± 0.02 ^cB^	2.62 ± 0.17 ^cC^	1.26 ± 0.06 ^cB^	89.75 ± 0.66 ^cB^
	B flour	0.39 ± 0.03 ^eB^	0.19 ± 0.01 ^dA^	1.01 ± 0.04 ^dC^	1.09 ± 0.06 ^dA^	48.57 ± 1.03 ^dA^
	R flour	0.70 ± 0.04 ^dB^	0.19 ± 0.01 ^dA^	0.98 ± 0.03 ^dC^	0.77 ± 0.03 ^eA^	42.35 ± 0.99 ^eB^
	Fine bran	3.89 ± 0.04 ^aA^	0.66 ± 0.04 ^bB^	14.41 ± 0.73 ^bA^	2.06 ± 0.09 ^bC^	95.50 ± 3.35 ^bB^
	Coarse bran	1.10 ± 0.02 ^cB^	4.84 ± 0.07 ^aB^	18.17 ± 1.12 ^aC^	7.60 ± 0.41 ^aA^	112.20 ± 0.42 ^aC^
JM26	Whole flour	1.93 ± 0.12 ^bA^	0.44 ± 0.00 ^cB^	4.96 ± 0.23 ^cA^	1.64 ± 0.09 ^cA^	115.10 ± 2.55 ^bA^
	B flour	0.88 ± 0.01 ^eA^	0.21 ± 0.01 ^dA^	3.20 ± 0.12 ^dA^	0.53 ± 0.03 ^dB^	30.04 ± 0.64 ^eB^
	R flour	1.19 ± 0.07 ^dA^	0.19 ± 0.00 ^eA^	3.29 ± 0.13 ^dA^	0.53 ± 0.03 ^dB^	59.06 ± 0.80 ^dA^
	Fine bran	2.65 ± 0.00 ^aC^	0.75 ± 0.00 ^bB^	13.80 ± 0.98 ^bA^	2.37 ± 0.11 ^bB^	78.07 ± 1.07 ^cC^
	Coarse bran	1.80 ± 0.01 ^cA^	3.56 ± 0.09 ^aC^	38.21 ± 1.82 ^aA^	7.38 ± 0.37 ^aA^	225.80 ± 7.92 ^aA^

Data are expressed as mean ± standard deviation (*n* = 3). Longtang 2 (LT2) was used as the control sample; Longtang 2 with foliar fertilization will be referred to as Longtang 2-Zn (LZ2); the other selected wheat Jimai26 (JM26) was a high-zinc variety. For the same parameter, means with different superscript lowercase letters within different milling fractions of the same wheat species are significantly different (*p* < 0.05), and means with different superscript uppercase letters within different wheat samples of the same milling fractions are significantly different (*p* < 0.05).

**Table 4:** Mineral composition (mg/kg) of milling fractions of LT2, LZ2, and JM26.

Wheat Sample	Fraction	Ca	Fe	Zn
LT2	Whole flour	343.52 ± 4.95 ^cB^	36.10 ± 0.85 ^cB^	28.81 ± 0.20 ^cB^
	B flour	245.81 ± 3.54d ^AB^	18.15 ± 0.21 ^dC^	8.58 ± 0.03 ^dC^
	R flour	222.39 ± 2.82 ^eB^	8.10 ± 0.18 ^eB^	7.60 ± 0.05 ^dC^
	Fine bran	510.36 ± 0.71 ^bB^	56.91 ± 0.14 ^bA^	52.72 ± 3.51 ^bB^
	Coarse bran	982.17 ± 18.38 ^aC^	111.72 ± 2.12 ^aC^	96.07 ± 0.82 ^aC^
LZ2	Whole flour	364.64 ± 2.12 ^cA^	39.05 ± 0.35 ^cA^	37.70 ± 0.13 ^cA^
	B flour	250.87 ± 9.19 ^dA^	24.65 ± 0.92 ^dA^	10.80 ± 0.07 ^dB^
	R flour	234.90 ± 6.36 ^eA^	8.77 ± 0.25 ^eB^	9.65 ± 0.02 ^dB^
	Fine bran	493.85 ± 16.26 ^bA^	58.33 ± 2.12 ^bA^	72.23 ± 0.66 ^bA^
	Coarse bran	1085.24 ± 7.07 ^aA^	138.58 ± 0.71 ^aA^	130.95 ± 1.06 ^aA^
JM26	Whole flour	365.60 ± 3.54 ^cA^	39.30 ± 0.71 ^cA^	37.62 ± 0.30 ^cA^
	B flour	240.83 ± 6.36 ^dB^	20.32 ± 0.42 ^dB^	11.57 ± 0.02 ^dA^
	R flour	208.33 ± 11.31 ^eC^	11.35 ± 0.64 ^eA^	10.92 ± 0.18 ^dA^
	Fine bran	492.69 ± 14.85 ^bA^	59.11 ± 1.98 ^bA^	74.64 ± 0.37 ^bA^
	Coarse bran	995.93 ± 6.36 ^aB^	132.42 ± 1.41 ^aB^	119.85 ± 1.06 ^aB^

Data are expressed as mean ± standard deviation (*n* = 3). Longtang 2 (LT2) was used as the control sample; Longtang 2 with foliar fertilization will be referred to as Longtang 2-Zn (LZ2); the other selected wheat Jimai26 (JM26) was a high-zinc variety. For the same parameter, means with different superscript lowercase letters within different milling fractions of the same wheat species are significantly different (*p* < 0.05), and means with different superscript uppercase letters within different wheat samples of the same milling fractions are significantly different (*p* < 0.05).

**Table 5:** Polyphenols and phytates contents, phytase activity, and mineral molar ratios of milling fractions of LT2, LZ2, and JM26.

Wheat Samples	Fraction	Polyphenols (mg/g)	Phytates (mg/g)	Phytase (U/g)	Phy:Ca	Phy:Fe	Phy:Zn
LT2	Whole flour	1.30 ± 0.01 ^cB^	15.22 ± 0.76 ^bA^	0.48 ± 0.03 ^cA^	2.69 ± 0.08 ^bA^	36.09 ± 2.73 ^aA^	52.50 ± 2.75 ^aA^
	B flour	1.26 ± 0.09 ^cA^	1.47 ± 0.04 ^cB^	0.27 ± 0.01 ^dA^	0.36 ± 0.03 ^dB^	6.88 ± 0.51 ^dA^	16.67 ± 1.28 ^cA^
	R flour	1.06 ± 0.04 ^dB^	1.23 ± 0.06 ^cA^	0.19 ± 0.01 ^eB^	0.33 ± 0.01 ^dA^	12.86 ± 0.77 ^cA^	15.91 ± 0.45 ^cA^
	Fine bran	1.56 ± 0.08 ^bA^	18.76 ± 0.79 ^bA^	0.59 ± 0.05 ^bA^	2.23 ± 0.02 ^cA^	27.96 ± 1.30 ^bA^	35.04 ± 1.32 ^bA^
	Coarse bran	2.03 ± 0.14 ^aA^	47.88 ± 4.71 ^aB^	0.80 ± 0.12 ^aB^	2.95 ± 0.11 ^aA^	36.36 ± 1.85 ^aA^	49.08 ± 0.66 ^aA^
LZ2	Whole flour	1.54 ± 0.17 ^bA^	15.99 ± 1.76 ^cA^	0.41 ± 0.05 ^cB^	2.66 ± 0.09 ^bA^	34.76 ± 0.62 ^aA^	41.78 ± 3.51 ^aB^
	B flour	1.20 ± 0.04 ^cA^	1.46 ± 0.09 ^dB^	0.27 ± 0.03 ^dA^	0.35 ± 0.01 ^cB^	5.02 ± 0.04 ^dB^	13.31 ± 0.63 ^cB^
	R flour	0.98 ± 0.07 ^dB^	1.15 ± 0.06 ^dA^	0.24 ± 0.02 ^dA^	0.30 ± 0.03 ^cA^	11.11 ± 0.89 ^cB^	11.72 ± 0.25 ^dB^
	Fine bran	1.66 ± 0.01 ^bA^	19.51 ± 1.50 ^bA^	0.54 ± 0.01 ^bA^	2.39 ± 0.24 ^bA^	28.42 ± 2.62 ^bA^	26.63 ± 1.89 ^bB^
	Coarse bran	2.09 ± 0.12 ^aA^	54.47 ± 2.89 ^aA^	0.87 ± 0.08 ^aA^	3.04 ± 0.17 ^aA^	33.41 ± 0.92 ^aB^	40.97 ± 3.32 ^aB^
JM26	Whole flour	1.40 ± 0.08 ^bAB^	12.47 ± 0.43 ^cB^	0.47 ± 0.03 ^cA^	2.07 ± 0.12 ^bB^	26.99 ± 1.99 ^aB^	32.64 ± 1.27 ^bC^
	B flour	1.33 ± 0.13 ^bcA^	1.70 ± 0.17 ^dA^	0.29 ± 0.01 ^dA^	0.43 ± 0.03 ^cA^	7.17 ± 0.56 ^cA^	14.49 ± 0.67 ^dB^
	R flour	1.28 ± 0.04 ^cA^	1.05 ± 0.17 ^dA^	0.25 ± 0.02 ^dA^	0.31 ± 0.01 ^dA^	7.95 ± 0.20 ^bC^	9.46 ± 0.08 ^eC^
	Fine bran	1.56 ± 0.05 ^bA^	17.43 ± 1.60 ^bA^	0.56 ± 0.03 ^bA^	2.14 ± 0.14 ^bA^	24.92 ± 0.39 ^aB^	22.97 ± 0.52 ^cC^
	Coarse bran	1.92 ± 0.04 ^aA^	39.33 ± 3.71 ^aC^	0.84 ± 0.11 ^aA^	2.39 ± 0.06 ^aB^	25.25 ± 1.73 ^aC^	35.26 ± 1.32 ^aC^

Data are expressed as mean ± standard deviation (*n* = 3). Longtang 2 (LT2) was used as the control sample; Longtang 2 with foliar fertilization will be referred to as Longtang 2-Zn (LZ2); the other selected wheat Jimai26 (JM26) was a high-zinc variety. For the same parameter, means with different superscript lowercase letters within different milling fractions of the same wheat species are significantly different (*p* < 0.05), and means with different superscript uppercase letters within different wheat samples of same milling fractions are significantly different (*p* < 0.05).

## Data Availability

Data is contained within the article.
